# Diet Quality Indices and Their Correlation with Glycemic Status and Lipid Profile in Patients with Type 2 Diabetes

**DOI:** 10.1155/2021/2934082

**Published:** 2021-03-06

**Authors:** Roxaneh Sadat Ziaee, Parisa Keshani, Moosa Salehi, Haleh Ghaem

**Affiliations:** ^1^School of Nutrition and Food Sciences, Shiraz University of Medical Sciences, Shiraz, Iran; ^2^Shiraz HIV/AIDS Research Center, Institute of Health, Shiraz University of Medical Sciences, Shiraz, Iran; ^3^Department of Epidemiology, School of Health, Shiraz University of Medical Sciences, Shiraz, Iran

## Abstract

**Background:**

The study aimed to determine the correlation between different diet quality indices and glycemic status and lipid profile in patients with diabetes.

**Methods:**

This study was carried out on 235 patients with type 2 diabetes referred to Martyr Motahari Diabetes Clinic of Shiraz city so as to investigate the relationship between glycemic status and lipid profile and the diet quality using Healthy Eating Index (HEI-2010), phytochemical index (PI), and Diet Quality Index-International (DQI_I).

**Results:**

A positive correlation was indicated between the serum levels of LDL-C and HEI-2010 scores (*p*=0.026). Furthermore, there was a positive correlation between the patients' age and scores of PI (*p*=0.006) and between PI and DQI_I (*p* < 0.001). There was no significant relationship between the scores for all three indicators and biochemical parameters.

**Conclusion:**

The results of this study indicated that there was a significant correlation between the serum LDL-C levels and the HEI-2010 scores. Moreover, the age of the patients had a direct correlation with the PI scores.

## 1. Introduction

Type 2 diabetes is a complex and chronic disease defined by a heterogeneous set of metabolic disorders including deficit in carbohydrate metabolism, protein, and lipid [1]. Nowadays, type 2 diabetes is one of the most challenging health problems around the world because of its high incidence, mortality caused by the disease, and the costs related to its treatment; there is a need for timely attention and monitoring in order to prevent the disease development and its better control [2]. Nutritional education and increase in nutritional awareness of patients can stop the developing process of type 2 diabetes or slow it down. Nowadays, various valid diet quality indices including Healthy Diet Indicator (HDI), Diet Quality Index (DQI), Alternate HEI (AHEI), Healthy Eating Index (HEI), DQI-International (DQI-I), and Mediterranean Diet Score have been introduced and developed [3]. Various studies have been conducted, indicating the correlation between diet quality and chronic diseases, especially cancer and cardiovascular diseases. Some researchers also have shown that there is a correlation between the quality indices and obesity and diabetes [4–7]. However, few studies are conducted on the correlation between diet quality indices and glycemic control and lipid profile; also, up to now, no studies have been carried out to evaluate the correlation between various indices. Diet quality indices might be used as a rapid evaluator of healthy nutritional diets after education of diet. With regard to the role of diet and/or medical nutrition treatment in chronic diseases, the approach of diet quality which includes adequate indices in diet is suggested as a powerful tool because of the complexity of diets. The HEI-2010 and the DQI-I are most common and used indices to assess diet in patients with noncommunicable diseases based on different items including macronutrients and micronutrients. Plant-extracted substances such as phytochemicals have been suggested to have antidiabetic activity [8]. Also, numerous studies have reported that diabetes mellitus causes more free radicals and decreases antioxidant parameters and capacity. Therefore, in some studies using phytochemicals is recommended to progress the insulin sensitivity [9]. Phytochemical index (PI) is an index to assess these compounds in foods [10]. In this way, the aim of the present study was to evaluate the correlation of three diet quality indices with glycemic status and lipid profile in patients with type 2 diabetes.

## 2. Materials and Methods

### 2.1. Participants

Using all three indices, the higher sample size was calculated by HEI considering SD = 12.5, *α* = 0.05, and *d* = 1.6 [11]. The subjects included 235 patients with type 2 diabetes and on oral-antidiabetic medications who were selected by convenient sampling method, from patients referred to Shiraz Motahari Diabetes Clinic. Inclusion criteria were known case of type 2 diabetes for one to 10 years, and age 30–65 years old. Patients who had diabetes related complications (neuropathy, nephropathy, or atherosclerosis), organ dysfunctions, recent surgery, and pregnancy and lactation or using immunosuppressive and immunoregulatory drug or insulin were excluded from the study. Firstly, the aims and stages of study were completely described for them and after justifying the participants, written consent forms were completed. This study was approved by ethical commission in Shiraz University of Medical Sciences no. IR.SUMS.REC.1394.S637.

### 2.2. Demographic, Anthropometric, and Clinical Parameters

Demographic information including gender (male, female), education (illiterate, primary school, secondary school, high school and diploma, and university), employment (unemployed and housekeeper, employed, and retired), and marital status (single, married, widow, and divorced), and their physical activity was asked of the participants. At least 150 to 300 minutes a week of moderate-intensity physical activity, or 75 to 150 minutes in a week of vigorous-intensity physical activity, is considered as physically active in this study [12].

Height was measured to the nearest centimeter, while the subjects were barefoot, and body weight was measured to the nearest 0.1 kg using a digital scale, while the subjects wore light clothing. Then, body mass index (BMI) was calculated by determining the ratio between weight and height squared (kg/m2). Blood pressure was recorded using a mercury sphygmomanometer twice in a sitting position after the subjects were in a relaxed state for at least 15 minutes. Again, after 5 minutes it was measured from the right arm and the average of two measurements were recorded as the final rate of blood pressure.

Venous blood samples (10 cc) were taken after 12 hours overnight fasting. The blood samples taken were immediately centrifuged and the serum was separated and maintained in −70 centigrade degrees up to the trial time. At the end of the study, the rates of glucose concentrations, insulin, glycosylated hemoglobin, triglyceride, total cholesterol, and HDL and LDL cholesterol were measured by commercial kits. Insulin and hemoglobin A_1_c were measured by Human Insulin ELISA kit (PARS AZMUN) and high-performance liquid chromatography kits (PARS AZMUN), respectively. Insulin resistance was calculated using the following formula:

insulin resistance = (fasting insulin (mIU/L) × fasting glucose (mmol/L))/22.5.

### 2.3. Dietary Assessment

Data about nutrition received by the patients were collected using the 24-hour dietary recall related to three days of the week (first day of week, midweek, and end of week). A 24-hour dietary recall is an organized interview, planned to collect information about all foods and beverages, ingredients, and the amount consumed by the respondent in the past 24 hours [13, 14].

On the visiting day, the first 24-hour recall was asked from the patients; also, the second and third reminders were asked from them by telephone and completed. The energy and macronutrients uptake were measured using the NUTRITIONIST-4 software and revised according to Iranian diet. With regard to not completing the table of Iranian compounds for the number of nutritional items and micronutrients, USDA table of nutritional compounds was also used in order to further analyze the nutritional items for receiving energy and nutrition. Common daily nutrition uptake of the patients was evaluated according to the household modules and then the daily received amounts were converted to gram. For mixed foods, the nutrition was also calculated according to the sum of the nutrition of the ingredients of that food. Finally, the data were used for measurement of the quality of the diet for each individual. Then, it was calculated according to HEI-2010, DQI-I, and PI indices, using the results of three 24-hour nutrition recall questionnaires.

### 2.4. Application of the HEI-2010, DQI-I, and PI to the Datasets

The HEI-2010 has 12 food components, 9 adequacy components with higher scores indicating higher consumption, and 3 moderation components with higher scores indicating lower consumption. The adequacy components include total fruit (5 points); whole fruit (5 points); total vegetables (5 points); greens and beans (5 points); whole grains (10 points); dairy (10 points); total protein foods (5 points); seafood and plant proteins (5 points); and fatty acids (10 points). The components that should be served in moderation include refined grains (10 points); sodium (10 points); and empty calories (solid fats, alcoholic beverages, and added sugars) (20 points). The mentioned scores were adjusted for energy (per 1,000 kcal or as a percentage of energy), except for the fatty acids ratio. The total HEI-2010 score which has a maximum value of 100 is a sum of all components, with higher total scores reflecting better diet quality [15].

The DQI-I focuses on 4 major aspects: variety (0–20 points), adequacy (0–40 points), moderation (0–30 points), and overall balance (0–10 points). The scores for all 4 categories were totaled, from 0 to 100 (0 being the worst and 100 the best possible scores) [16].

The dietary PI was calculated based on the method developed by McCarty [17] PI = [daily energy derived from phytochemical-rich foods kJ (kcal)/total daily energy intake kJ (kcal)]^*∗*^ 100. Foods included in the phytochemical-rich category were fruits and vegetables, legumes, whole grains, seeds, nuts, natural fruit and vegetable juices, olive oil, and soy products.

### 2.5. Statistical Analysis

Due to the normal distribution of data, the mean and standard deviation was used to describe quantitative data and the frequency and percentage for qualitative variables. We used Kolmogrov-Smirnov and Shapiro-wilk tests to check the normality and Pearson tests to evaluate the correlation between variables as needed. Considering the significance level of 0.05, SPSS software version 20 was used to analyze the data and NUTRITIONIST-4 software was used to evaluate the nutritional status and extraction of micronutrients and macronutrients.

## 3. Results

From the 235 patients, 148 (63%) were men and 87 (27%) were women. The mean age of the patients was 55.11 ± 7.89 years. The average values of height, weight, body mass index, blood pressure, and metabolic indices of patients are shown in Table 1. The results showed that there was a significant positive correlation of PI with age and HDL; on the other hand, there was a significant reverse correlation between BMI of patients and PI. There was no significant correlation between the variables evaluated with DQI-I index. However, total cholesterol levels and LDL-C levels of patients had a direct and significant correlation with HEI-2010 score (Table 2). The mean scores for DQI-I, HEI-2010, and PI in this study were 58.82 ± 7.34 (Table 3), 55.83 ± 10.61 (Table 4), and 36.79 ± 15.99.

## 4. Discussion

The evaluation of diet based on diet quality indices offers us a more comprehensive approach about the correlation of nutrition receiving and health, compared to evaluation of receiving nutrition only. Inappropriate diet quality and absence of physical activity have led to increase in the obesity incidence and thereupon type 2 diabetes in developed and developing countries [18]. Also, it has been observed that high-quality diet is related to a decrease in the mortality risk caused by cardiovascular diseases and a decrease in the risk of type 2 diabetes [19]. Type 2 diabetes is one of the most challenging health problems around the world which needs timely attention and follow-up in order to prevent the development of the disease and its better control. The evaluation of the diet quality and recognition of appropriate index related to better glycemic control and better status of lipid profile can be used for presentation of applied and effective nutritional recommendations in order to remove the existing nutritional problems. In Iran, a limited number of studies have been conducted on diet quality indices, especially in diabetic patients, and the three indices of diet quality (DQI-1, HEI-2010, and PI) have been evaluated in none of these studies.

In the present study, there was only a direct correlation between the patients' age and the score of phytochemical index and score of HEI-2010 index; this correlation was statistically significant only with phytochemical index (*p*=0.006). Also, in other studies, a significant correlation has been reported between age and diet quality indices. For instance, in Mariscal-Arcas's study, there was also a significant correlation between DQI-I and age [20]. Also, in Coltman's study, it was observed that older people had a higher HEI score compared to the patients between 40 and 60 years old. Their results were consistent with ours [21]. Also, in a study carried out by Patricia Guenther, the mean score of HEI-2010 was lower in younger adults compared to older adults; the obtained results were similar to both editions of HEI (HEI-2005 and HEI-2010) [22]. In the studies conducted in Iran, it has also been observed that older participants have higher phytochemical indices [23, 24] although few studies have revealed different results. This is similar to Mabililoni's study, in which reverse and significant correlation was observed between age and DQI-I score [25]. Therefore, it can be stated that increase in age is correlated with better adherence to nutritional recommendations. One of the possible reasons can be the necessity of the control of chronic diseases present at these ages due to adherence to the diet recommendations which leads to improvement of the quality of diet with increase in age.

There was no significant statistical correlation between the anthropometric indices and blood pressure of type 2 diabetes patients and diet quality assessment indices, in the present study, which were parallel with results of Mariscal-Arcas study [20]; why it is so in this study can be illustrated in that the obesity status and body mass index did not show a significant correlation with DQI-I [25] although the evaluation of DQI-I components was effective in the correlation shown between obesity status and BMI with DQI-I.

However, in the study of Nicklas, it was observed that BMI, waist perimeter, diastolic blood pressure, and score of HEI-2005 index had a reverse correlation. Also, individuals who had the highest diet quality were less affected with obesity or overweight, increase of waist perimeter, and high blood pressure [26]. In the study of Quatromoni, women and men with the highest DQI score had also a lower weight [27]. In fact, the results of this study indicate favorable effects of antioxidants on metabolic disorders, prevention of reweighing, and increase of abdominal obesity [28]. Furthermore, in the study of Mirmiran, the scores higher than phytochemical index had a reverse correlation with 3-year alteration of weight, waist perimeter, and body fat [29]. Also, high intake of fruits was correlated with a decrease in overweight. In fact, high score of phytochemical index had favorable effects on prevention of reweighing and decrease of fat in adults. In another study, the participants with higher PI had lower weight and waist perimeter and the risk of abdominal obesity was 66% lower among them. As a result, it has been observed that high intake of foods rich in phytochemicals had a reverse correlation with abdominal obesity.

### 4.1. The Correlation between Glycemic Status and Diet Quality Evaluation Indices

In the present study, which was a sectional study for evaluation of the correlation between diet quality and glycemic status in patients with type 2 diabetes, no significant correlations were observed between the scores of the 3 evaluated indices, fasting blood sugar, glycosylated hemoglobin (HbA1c), insulin, and insulin resistance. It is noteworthy that there was a high coherence between the two variables of insulin and insulin resistance (*p* < 0.001 and *r* = 0.901); therefore, the independent variable of insulin was replaced with resistance to insulin. Few studies have evaluated the correlation of diet quality and fasting blood sugar and insulin, and most of them have evaluated the correlation of the risk of diabetes and scores of diet quality indices. In the study conducted by Juturu (2006), the serum insulin levels and insulin resistance had a significant correlation with HEI scores in the patients with type 2 diabetes [30]. In another study by Chiuve (2012), there was a low correlation between AHEI-2010 and HEI-2005 with coronary artery diseases and type 2 diabetes. The results of this study showed that adherence to the HEI-2005 recommendations might decrease the risk of chronic diseases, but AHEI-2010, which includes more nutritional information, had a higher correlation with the risk of chronic diseases, especially CHD and diabetes [31]. In the study of Ferranti, higher AHEI score had also a reverse correlation with HbA1C, indicating the protective effect of adherence to AHEI recommendations on cardiometabolic factors in women with a history of GDM (pregnancy diabetes) [32]. Also, in another study, similar results were observed and the incidence of fasting blood sugar disorder was decreased by 75% in men with higher HEI scores [7]. Further, consumption of mixed diets of fruits and vegetables had a high reverse correlation with the incidence of type 2 diabetes; these findings indicate the importance of consumption of fruits and vegetables in prevention of type 2 diabetes [33].

The results of the present study indicated a direct correlation between serum LDL-C levels and the score of HEI-2010 index and DQI-I index which had only significant correlation with HEI-2010 index (*p*=0.026). In the study carried out by Nicklas, HEI-2005 score had a reverse correlation with total cholesterol and LDL and a direct and significant correlation with HDL-C. Furthermore, people with the highest diet quality were less affected with decrease in HDL [26]. In another study, HEI-2005 index was applied for evaluation of the quality of diet, and it was observed that people with higher percentile of HEI had lower LDL and triglyceride and higher HDL [34].

Studies performed in Iran also reported similar results. In a study conducted by Asghari et al., it was reported that men with the highest score of HEI-2005 index had significantly lower levels of serum triglyceride although this correlation was not seen among women. On the other hand, a direct correlation was observed in men between DQI-1 and HDL-C scores. Moreover, in both genders, all three indices had a weak correlation with total cholesterol, while no correlation was observed between the indices with LDL-C. In fact, HEI-2005 index had a weak correlation with a decrease in serum levels of triglyceride in urban population [10]. According to animal and human studies, it has been reported that herbal polyphenols in diet and consumption of products rich in polyphenols lead to adjustment of carbohydrate and fat metabolism, decrease in the level of sugar, decrease in disorders of blood fat, reduction of insulin resistance, improvement of adipose tissue metabolism, and decrease in the oxidative stress and inflammation. Polyphenolic contents can prevent development of chronic complications of diabetes including cardiovascular diseases, nephropathy, neuropathy, and retinopathy [35].

While most of the previous studies had reported a reverse and significant correlation between diet quality and serum levels of LDL-C, the results of the present study indicated a direct and significant correlation between them; it seems that the reason for the contradiction about the correlation between the score of diet quality index and lipid profile with previous studies is that, in the present study, most patients consumed blood fat decreasing drugs such as atorvastatin that this might have been a confounding factor as to this correlation. On the other hand, in the present study, the duration of having diabetes in participants was not considered, while this can be one of the other confounding factors. Also, over time and due to better control of disease and its complications, the participants directed their diets into having a more healthy diet. Also, the nature of type 2 diabetes is the other reason for justifying the increase in the serum levels of LDL-C in patients participating in the present study. It is noteworthy that, in previous studies, most of the communities evaluated had been healthy people, while in the present study the subjects were patients with type 2 diabetes.

In the present study, PI, HEI-2010 and DQI-I indices had a weak correlation with each other and only the correlation between PI and DQI-I indices was significant (*p* < 0.001 and *r* = 0.232).

Very limited studies have evaluated diet quality indices simultaneously. Therefore, the available results are few in order to evaluate and compare them with the results of the present study. In one of these limited studies, although a direct correlation was seen in men between scores of DQI-I and HDL-C, in both genders, all the three indices (HEI-2005, MDS, and DQI-I) had a weak correlation with total cholesterol. However, no correlation was observed between the indices with serum LDL-C. Only HEI-2005 index had a weak correlation with the decline in serum triglyceride concentrations among the urban population of Iran, but there was no correlation between the two indices of MDS and DQI-I with lipid profile. Although, in this study, the correlation between indices was not evaluated directly, the results of the study indicated a weak correlation between these indices. Therefore, it seems that the intended indices for diet quality evaluation have a weak and insignificant correlation with each other. Furthermore, since the present study is the only study reporting the significant correlation of PI and DQI indices, it is essential to conduct more studies to confirm this aspect.

Our study had some limitations including low sample size, no long-term follow up of the patients, and no consideration of the duration of diabetes disease and drug regimens in patients as a confounding factor.

## 5. Conclusions

Generally, it seems that inconsistency of the findings in the present study with some previous studies is due to the difference in nutritional habits and type and amount of meals among various countries. Why it is so even in similar amounts is that the quality and nutritional values are not similar and also the diet quality evaluation indices are formed based on the information of societies which have numerous differences with our population of the study.

## Figures and Tables

**Table 1:** Demographic and clinical characteristics of participants (*n* = 235).

Variables	Mean ± SD
Age	55.11 ± 7.89
BMI	27.02 ± 3.66
BP-systolic	127.38 ± 15.30
BP-diastolic	81.52 ± 7.19
TG (mg/dl)	193.96 ± 79.54
Insulin (*µ*U/ml)	8.60 ± 9.45
TC (mg/dl)	163.61 ± 39.94
LDL-C (mg/dl)	92.75 ± 28.25
HDL-C (mg/dl)	45.72 ± 10.44
HA1C (%)	7.80 ± 1.55
Insulin resistance	3.28 ± 4.71
FBS (mg/dl)	148.13 ± 62.57

	n%
*Gender*	
Female	148 (63.0)

*Marital status*	
Single	9 (3.8)
Married	215 (91.5)
Widow and divorced	11 (4.7)

*Education*	
Illiterate	2 (0.9)
Primary school	65 (27.7)
Secondary school	51 (21.7)
High school and diploma	77 (32.8)
University	40 (17.0)

*Employment*	
Unemployed and housekeeper	135 (57.4)
Employed	52 (22.1)
Retired	48 (20.4)

*Physically active*	
Yes	92 (39.1)

BMI, body mass index; BP, blood pressure; FBS, fasting blood sugar; HbA1c, hemoglobin A1c; HDL, high-density lipoprotein cholesterol; LDL-C, low-density lipoprotein cholesterol; TG, triglyceride; TC, total cholesterol.

**Table 2:** Correlation (*r*, *p* value) between age, BMI, and clinical/biochemical variables and three diet quality indices.

Diet quality indices		
Variables *r* *p* value	DQI-I	HEI	PI
Age	0.062	0.104	0.154
	0.345	0.111	0.018*∗*

BMI	−0.22	−0.123	−0.131
	0.740	0.059	0.045*∗*

BP-systolic	−0.023	0.002	−0.032
	0.729	0.972	0.629

BP-diastolic	−0.062	0.11	−0.095
	0.348	0.869	0.146

TG (mg/dl)	0.006	0.110	−0.098
	0.931	0.094	0.136

Insulin (*µ*U/ml)	−0.034	−0.050	−0.098
	0.602	0.449	0.135

TC (mg/dl)	0.083	0.110	−0.014
	0.207	0.039*∗*	0.833

LDL-C (mg/dl)	0.089	0.130	-0.046
	0.174	0.047*∗*	0.485

HDL-C (mg/dl)	0.046	0.058	0.129
	0.485	0.378	0.048*∗*

HbA1c (%)	0.017	0.49	−0.002
	0.790	0.453	0.977

Insulin resistance	0.064	0.113	0.093
	0.801	0.884	0.102

FBS (mg/dl)	−0.86	0.035	−0.090
	0.189	0.591	0.171

^*∗*^ *p* < 0.05. BMI, body mass index; BP, blood pressure; DQI-I, Diet Quality Index-International; FBS, fasting blood sugar; HEI, Healthy Eating Index; HbA1c, hemoglobin A1c; HDL, high-density lipoprotein cholesterol; LDL-C, low-density lipoprotein cholesterol; PI, phytochemical index; TG, triglyceride; TC, total cholesterol.

**Table 3:** DQI-International (DQI-I) components and criteria used for maximum score and zero score (*n* = 235).

Components	Score	Scoring criteria	Mean ± SD
DQI-I, total	0–100	—	58.2 ± 7.34

***Variety***	0–20	—	19.77 ± 0.77
Overall food group variety	0–15	≥1 serving from each food group/d = 15	14.88 ± 0.64
		Any 1 food group missing/d = 12	
		Any 2 food groups missing/d = 9	
		Any 3 food groups missing/d = 6	
		≥4 food groups missing/d = 3; none = 0	

Within-group variety for protein source	0–5	≥3 different sources/d = 5; 2 different sources/d = 3; from 1 source/d = 1; none = 0	4.88 ± 0.46

***Adequacy***	**0–40**		25.98 ± 5.53
Vegetable group	0–5	≥3–5 servings/d = 5; 0 servings/d = 0	1.42 ± 2.26
Fruit group	0–5	≥2–4 servings/d = 5; 0 servings/d = 0	1.00 ± 2.01
Grain group	0–5	≥6–11 servings/d = 5; 0 servings/d = 0	4.03 ± 1.98
Fiber	0–5	≥20–30 g/d = 5; 0 g/d = 0	3.53 ± 2.28
Protein	0–5	≥10% of energy/d = 5; 0% of energy/d = 0	4.90 ± 0.10
Iron	0–5	≥100% of RDA(AI)/d = 5; 0% of RDA(AI)/d = 0	4.98 ± 0.26
Calcium	0–5	≥10% of AI/d = 5; 0% of AI/d = 0	4.97 ± 0.32
Vitamin C	0–5	≥100% of RDA(RNI)/d = 5; 0% of RDA(RNI)/d = 0	4.70 ± 0.80

***Moderation***	**0–30**		12.45 ± 4.35
Total fat	0–6	≤20% of total energy (TE)/d = 6; >20–30% of TE/d = 3; >30% of TE/d = 0	3.13 ± 2.09
Saturated fat	0–6	≤7% of TE/d = 6; >7–10% of TE/d = 3; >10% of TE/d = 0	1.06 ± 0.71
Cholesterol	0–6	≤300 mg/d = 6; >300–400 mg/d = 3; >400 mg/d = 0	5.41 ± 1.48
Sodium	0–6	≤2,400 mg/d = 6; >2,400–3,400 mg/d = 3; >3,400 mg/d = 0	0.87 ± 1.80
Empty calorie foods	0–6	3% of TE/d = 6; >3–10% of TE/d = 3; >10% of TE/d = 0	3.04 ± 2.47

***Overall balance***	**0–10**		0.61 ± 1.71
Macronutrient ratio	0–6	55–65 : 10–15 : 15–25 = 6; 52–68:9–16 : 13–27 = 4; 50–70:8–17 : 12–30 = 2; otherwise = 0	0.25 ± 1.21
Fatty acid ratio	0–4	P/S = 1–1.5 and M/S = 1–1.5 = 4; else if P/S = 0.8–1.7 and M/S = 0.8–1.7 = 2; otherwise = 0	0.36 ± 1.11

AI, adequate intake; d, day; M, monounsaturated fatty acid; P, polyunsaturated fatty acid, RDA, Recommended Dietary Allowances; S, saturated fatty acid; TE, total energy.

**Table 4:** Healthy Eating Index (HEI-2010) components and criteria used for maximum score and zero score (*n* = 235).

Components	Score	Scoring criteria	Mean ± SD
HEI, total score	0–100		55.83 ± 10.61

***Adequacy***	**0–60**		12.71 ± 1.12
Total fruit	0–5	≥0.8 cup equiv. per 1,000 kcal; no fruit = 0	1.23 ± 0.94
Whole fruit	0–5	≥0.4 cup equiv. per 1,000 kcal; no whole fruit = 0	0.92 ± 0.70
Total vegetables	0–5	≥1.1 cup equiv. per 1,000 kcal; no vegetables = 0	1.85 ± 1.16
Greens and beans	0–5	≥0.2 cup equiv. per 1,000 kcal; no dark green vegetables or beans and peas = 0	0.24 ± 0.26
Whole grains	0–10	≥1.5 oz equiv. per 1,000 kcal; no whole grains = 0	1.55 ± 1.90
Dairy	0–10	≥1.3 cup equiv. per 1,000 kcal; no dairy = 0	0.35 ± 0.31
Total protein foods	0–5	≥2.5 oz equiv. per 1,000 kcal; no protein foods = 0	2.96 ± 2.24
Seafood and plant proteins	0–5	≥0.8 oz equiv. per 1,000 kcal; no seafood or plant proteins = 0	1.18 ± 1.59
Fatty acids	0–10	(PUFAs + MUFAs)/SFAs ≥2.5; (PUFAs + MUFAs)/SFAs ≤1.2	2.42 ± 0.80

***Moderation***	**0–40**		6.15 ± 1.21
Refined grains	10	≤1.8 oz equiv. per 1,000 kcal; ≥4.3 oz equiv. per 1,000 kcal	5.04 ± 3.47
Sodium	10	≤1.1 grams per 1,000 kcal; ≥2.0 grams per 1,000 kcal	0.90 ± 1.01
Empty calories	20	≤19% of energy; ≥50% of energy	0.11 ± 0.17

HEI, Healthy Eating Index; MUFA, monounsaturated fatty acid; PUFA, polyunsaturated fatty acid; SFA, saturated fatty acid.

## Data Availability

Data are available upon request to the corresponding author.
